# Training and proficiency level in endoscopic sinus surgery change residents’ eye movements

**DOI:** 10.1038/s41598-022-25518-2

**Published:** 2023-01-03

**Authors:** Laura Niederhauser, Sandra Gunser, Manuel Waser, Fred W. Mast, Marco Caversaccio, Lukas Anschuetz

**Affiliations:** 1 Department of Psychology, University of Bern, Bern, Switzerland; 2 Department of Otorhinolaryngology, Head and Neck Surgery, Inselspital, University Hospital Freiburgstrasse 18, University of Bern, 3010 Bern, Switzerland

**Keywords:** Health policy, Prognosis, Public health, Health care, Medical research

## Abstract

Nose surgery is challenging and needs a lot of training for safe and efficient treatments. Eye tracking can provide an objective assessment to measure residents’ learning curve. The aim of the current study was to assess residents’ fixation duration and other dependent variables over the course of a dedicated training in functional endoscopic sinus surgery (FESS). Sixteen residents performed a FESS training over 18 sessions, split into three surgical steps. Eye movements in terms of percent fixation on the screen and average fixation duration were measured, in addition to residents’ completion time, cognitive load, and surgical performance. Results indicated performance improvements in terms of completion time and surgical performance. Cognitive load and average fixation duration showed a significant change within the last step of training. Percent fixation on screen increased within the first step, and then stagnated. Results showed that eye movements and cognitive load differed between residents of different proficiency levels. In conclusion, eye tracking is a helpful objective measuring tool in FESS. It provides additional insights of the training level and changes with increasing performance. Expert-like gaze was obtained after half of the training sessions and increased proficiency in FESS was associated with increased fixation duration.

## Introduction

Functional endoscopic sinus surgery (FESS) are complex operations and place high demands on the surgeon's skills and precision^1^. The endoscope is navigated through the challenging anatomy of the nasal cavity and paranasal sinuses with one hand, and surgery is usually performed with the other. The instruments and surgical environment cannot be seen directly, but are visualised on a screen. In addition, depth perspective is missing with 2-dimensional endoscopes. These peculiarities have a negative influence on the learning curve. A lot of training is necessary to master the eye-hand coordination of these surgical techniques efficiently and safely^2^. Moreover, the three-dimensional anatomy of the paranasal sinus and the relative proximity of vital structures make a thorough training mandatory for young surgeons.

The education of surgical skills in FESS relies mainly on cadaveric training courses, use of simulators and teaching inside the operating room. The assessment of when a trainee is ready for the next step or first unsupervised steps is usually dependent on the senior surgeon’s subjective evaluation. However, the implementation of objective measures such as eye tracking would be an additional tool in the assessment of surgical expertise. Eye tracking measures the surgeons’ gaze behaviour during a procedure with head-mounted glasses. One important measurement in eye tracking is fixation duration. A fixation refers to the moment when the eyes are focusing on a certain location, enabling the viewer to perceive visual acuity and indicating cognitive processing^3–5^ Fixations have different functions; in a surgical simulation, they are mainly used to guide the surgeons’ movements and check the relevant areas or objects in their environment^6^. This is crucial for effective hand movements and a proficient performance, since hand and eye motion are interchangeably connected^7–9^. The movements between fixations are called saccades. During saccades, visual acuity is reduced. Therefore, surgeons want to minimize these movements and apply an efficient gaze to improve their performance. This means that with more experience they look around less and more on important areas, and they show longer fixations. Research in different surgical specialities has been able to show this learning curve, which leads to different eye movements in experienced and novice surgeons^10^. In laparoscopic surgery, consultants spent more time fixating on the target than on surgical tools when compared to novices^11,12^. Results in neurosurgery and endoscopic ear surgery showed that expert surgeons apply a steadier gaze than novices, indicated by longer fixation durations^13,14^. In FESS, Ahmidi et al.^15^ proposed a statistical model to separate surgeons by their skill level based on their eye and hand movements. However, because they mixed motion and eye tracking measures, there were no insights into surgeons’ specific gaze behaviour.

Our aim was therefore to implement a practical surgical training for residents in FESS, whilst measuring their eye movements, completion time, perceived cognitive workload, and overall surgical performance. We hypothesized the correlation of eye movements related to surgical performance and experience. In addition, we implemented basic gaze instructions for half of the participants. Previous studies have shown potential for this kind of trainings^16–18^, therefore, we showed an experts’ gaze to half of the participants before their own training.

## Methods

### Sample

Sixteen residents of the Otorhinolaryngology, Head and Neck Surgery (ORL-HNS) Department of Inselspital, University Hospital Bern, Switzerland participated in this study. All methods were performed in accordance with the relevant guidelines and regulations. Based on our power analysis, sixteen participants are sufficient to detect effects of medium size with a power of 0.80. Participants received a financial refund at the end of the study. The ethical committee of the University of Bern granted approval to perform the study (2021-05-00004) and all participants gave written informed consent to participate before starting with the training program and study.

### Stimuli

All participants performed 18 sessions of a FESS training course over the course of several weeks from June to November 2021. To perform the surgeries, 4 mm diameter and 18 cm length rod lens endoscopes coupled to a high-definition camera system and monitor (Karl Storz, Tuttlingen, Germany) and standard tools for FESS were used. Participants held the tool in one hand and the camera in the other, aiming it at the relevant areas. We used a PHACON Sinus Trainer with PHACON Sinus Patient “Meyer” (normal anatomy) as specimen models for participants to perform the surgeries in a standardized environment (PHACON GmbH, Leipzig, Germany).

### Apparatus

To track participants’ eye-movements during the surgeries, we used eye-tracking glasses by Pupil Labs (Berlin, Germany)^19^. The eye-tracker sampled with 120 Hz and was connected to a computer running macOS 10.14 (Apple Inc., Cupertino, California) and Pupil Labs software (https://docs.pupil-labs.com/core/). Marker calibration was performed before each training session.

We defined the video screen as area of interest and assessed participants’ number of fixations placed on the screen in relation to the total amount of fixations made during the surgery (percent fixations on screen). In addition, we assessed their average duration of fixations placed on the screen in milliseconds (ms).

Completion time in minutes was measured for each session. After every session, participants indicated their perceived cognitive load during the surgery with the NASA-TLX^13,20–22^, a self-report measurement consisting of six subscales, each ranging from 0 to 100.

A highly experienced rhinologic surgeon blindly rated the first and last session of each step (sessions 1, 6, 7, 12, 13, and 18) separately by viewing the video recording. We used the average global rating of the objective structured assessment of technical skills score (OSATS^23^), adapted to endoscopic sinus surgery^24^.

At the beginning of the first session, measures such as demographics and experience in terms of performed surgeries in endoscopy were collected. Moreover, after each session participants indicated if their experience had increased. All questionnaires were administered via Qualtrics (www.qualtrics.com).

### Procedure

The endoscopic sinus surgeries consisted of three steps with gradually increasing complexity of the tasks. Taken together, all three steps would result in a functional ethmoidectomy. Participants had to perform each step six times before continuing with the next step, resulting in 18 sessions total. The surgical steps are described below:

1. Maxillary antrostomy: An endonasal endoscopic approach using the nostril as surgical access to the paranasal sinus was used. First, nasal endoscopy and study of the nasal cavity with identification of nasal septum, inferior and middle turbinate, middle meatus, uncinate process, ethmoid bulla, agger nasi area and olfactory cleft is performed. The middle turbinate is then gently mobilised medially, to gain access to the paranasal sinus system. The first step consists in the resection of the uncinated process, this step allows access to the maxillary sinus and later the anterior ethmoid. During resection of structures in the paranasal sinuses a continuous visual control is essential to avoid injury to adjacent structures (e.g., orbit, skull base) and to avoid tearing the mucosa from the underlaying bone. As next step, the identification of the natural ostium to the maxillary sinus and its inclusion into the antrostomy is crucial. The natural maxillary sinus ostium is expanded posteriorly and inferiorly to a size of approximatively 2 cm (Type II sinusotomy).
2. Anterior ethmoidectomy: From the previously performed maxillary antrostomy, the floor of the orbit can be localized. Accordingly, identification and opening the anterior inferior portion of the ethmoid bulla is performed, followed by its complete resection. Progressive resection of the anterior ethmoidal cells until the basal lamella is identified. The remaining mucosa is preserved. Identification of the lamina papyracea and resection of the agger nasi cell and completion of ethmoidectomy with identification of the frontal recess. In this step it is crucial to respect the orbit from surgical injury.
3. Posterior ethmoidectomy: Removal of the vertical portion of the basal lamella and opening of the posterior ethmoid using trough-cut instruments. The posterior ethmoidal cells are now removed until reaching the anterior wall of the sphenoid sinus. The identification of the skull base and lamina papyracea from posteriorly are most eminent to allow the preservation of these structures. Cleaning of the lamina papyracea and skull base from protruding septations from posterior to anterior.

**Figure 1:**
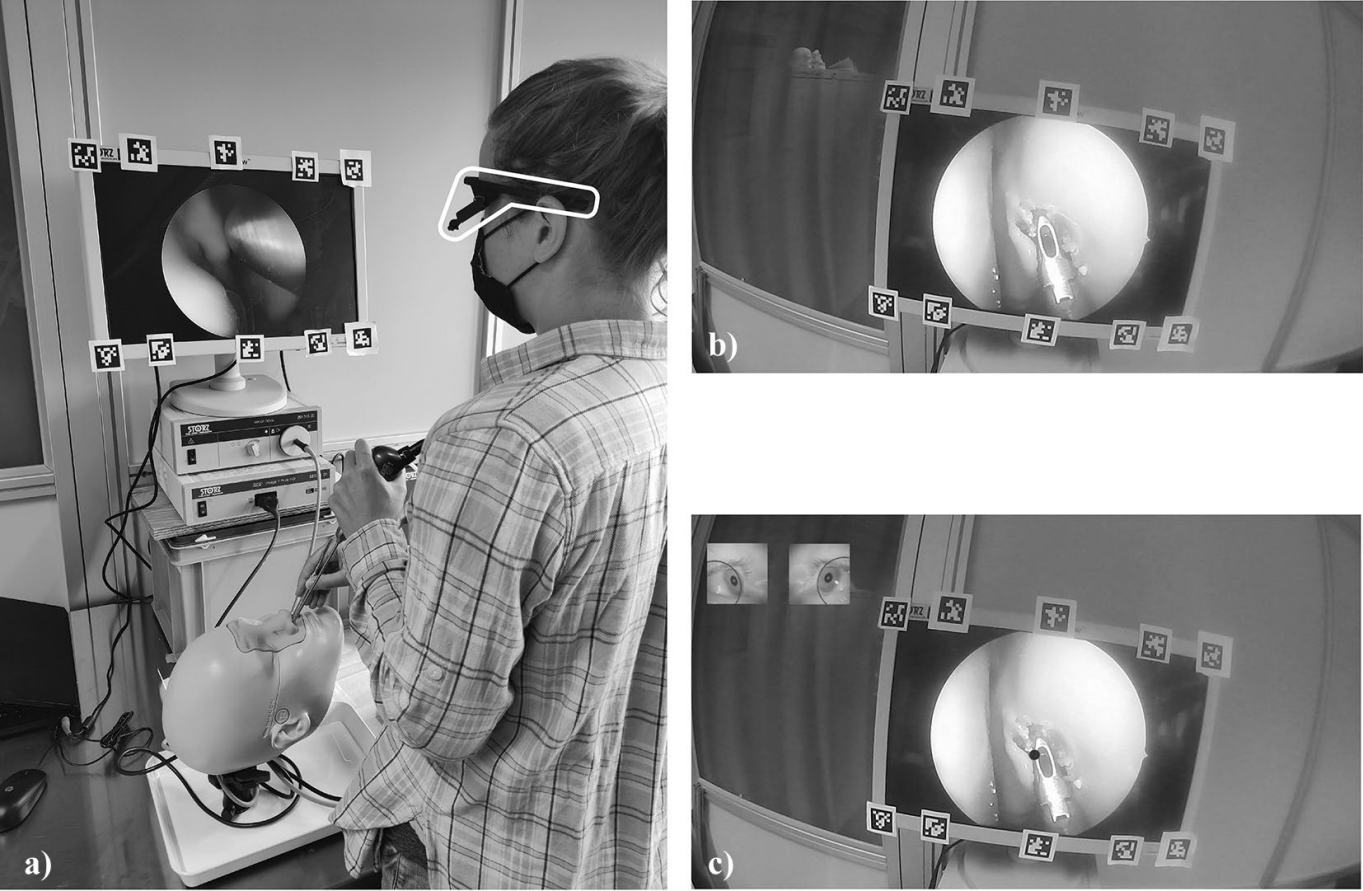
Study setup and instruction videos. (**a**) Participant performing endoscopic surgery. Eye movements are tracked with head mounted glasses, highlighted by a white frame. Black-white markers on the screen are used for the analysis of areas of interest. (**b**) Screenshot of the teaching video without gaze and (**c**) with gaze instruction.

Before each surgery, participants watched the video of an expert surgeon carrying out the same step with the same setup. The most important surgical stages were described in written form during the videos. No further instruction was given. During this video, half of participants did also see where the expert surgeon was looking during his performance, the other half did not see the gaze, see Fig. 1. We assured that participants in both groups had the same level of experience.

### Statistical analysis

We used frequencies and percentages to describe categorical variables, and means, medians and range for continuous variables. We used a natural log transformation to reduce skewness of average fixation duration and completion time.

Due to technical difficulties, data points for cognitive load (one missing) and eye movements (two missing) were missing. We treated them as completely random (MCAR) and applied a multiple imputation with chained equations (MICE) based on the two-level linear model method^25,26^. The MICE tool was found to be effective for imputation of missing longitudinal data in previous research^27,28^.

Analysis of the effect of sessions and group on dependent variables (average global rating, completion time, cognitive load, percent fixations on screen, average fixation duration) was done with a linear mixed model. Linear mixed models are like a general application of ANOVAs (analysis of variance) but do not have to satisfy assumptions like homoschedasticity and sphericity^29^. Degrees of freedom are approximated with the Satterthwaite’s method^30^. For explorative analysis of dependent variables, we used Welch’s t tests. Statistical analyses were conducted in R, version 4.1.0^31^.

## Results

A summary of participants’ demographic data is provided in Table 1. There were six (37.5%) female participants overall. Welch t-test revealed no statistically significant differences between the two groups regarding gaze instruction during the teaching videos. Four participants in each group had performed three or fewer surgeries before they started with the training.

**Table 1:** Overview of participants.

	Overall	Group with gaze	Group without gaze	Welch t-test
Age in years (M, range)	31.0 (26–39)	30.2 (26–35)	31.8 (26–39)	*p* = 0.419
Days between sessions (M, range)	4.22 (1–19)	4.04 (1–19)	4.40 (1–16)	*p* = 0.349
Total days for all 18 sessions (M, range)	71.75 (26–103)	68.6 (26–103)	74.9 (49–92)	*p* = 0.588
FESS experience (M, range, median)	20 (0–200), 4.5	30 (0–200), 4	10 (0–25), 6.5	*p* = 0.953
Performed surgeries during data collection (M, range, median)	0.27 (0–6), 0	0.19 (0–6), 0	0.35 (0–4), 0	*p* = 0.084
Year of residency (M, range)	3.5 (1–5)	3.25 (1–5)	3.75 (1–5)	*p* = 0.559

### Comparison between groups with and without gaze instruction

**Figure 2:**
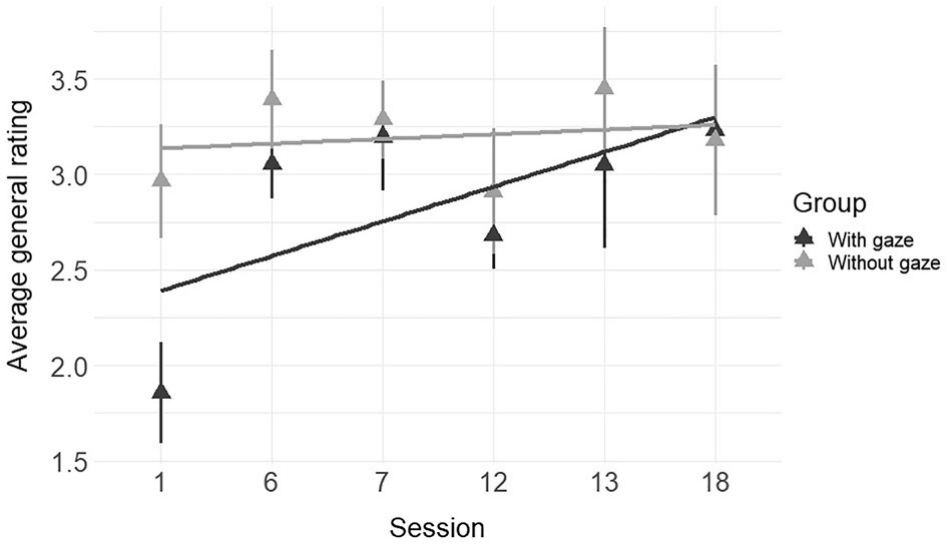
Surgical performance development for groups with and without gaze. Triangles depict the mean and vertical lines depict the standard error for the mean. The horizontal lines represent the linear effect of sessions.

Average global ratings of the technical skill score (OSATS) ranged from 1.14 to 5.0 on the 5-point scale, with a high internal consistency for the individual items (Cronbach’s *alpha* = 0.89). Analysis showed a statistically significant effect of the linear term for session number, *F*(1, 51) = 4.71, *p* = 0.035 and for group, *F*(1, 42) = 5.74, *p* = 0.021. There was a clear increase of ratings over sessions (mean change per session = 0.18 points; 95% CI: 0.05 to 0.31). The group without gaze instructions received overall higher ratings than the group with gaze instructions (mean difference = 0.90 points; 95% CI: 0.16 to 1.64). Figure 2 shows that this effect might be largely based on the lower rating in the first session. The interaction between group and session did not reach statistical significance though (95% CI: − 0.34 to 0.03). There was no further effect of gaze instruction on any other variable (supplemental material). Therefore, the following results are an analysis of both groups together.

### Correlations between dependent variables

Average global OSATS ratings correlated positively with participants’ previous FESS experience (*r* = 0.21, *p* = 0.044, 95% CI: 0.006 to 0.39). The correlation with completion time did not reach statistical significance level (*p* = 0.058), but was considerable (*r* =  − 0.19, 95% CI: − 0.38 to 0.006) ^32^. A table showing all possible correlations within the dependent variables can be found in the supplemental data (eTable 1).

### Development of dependent variables for the entire sample

**Figure 3:**
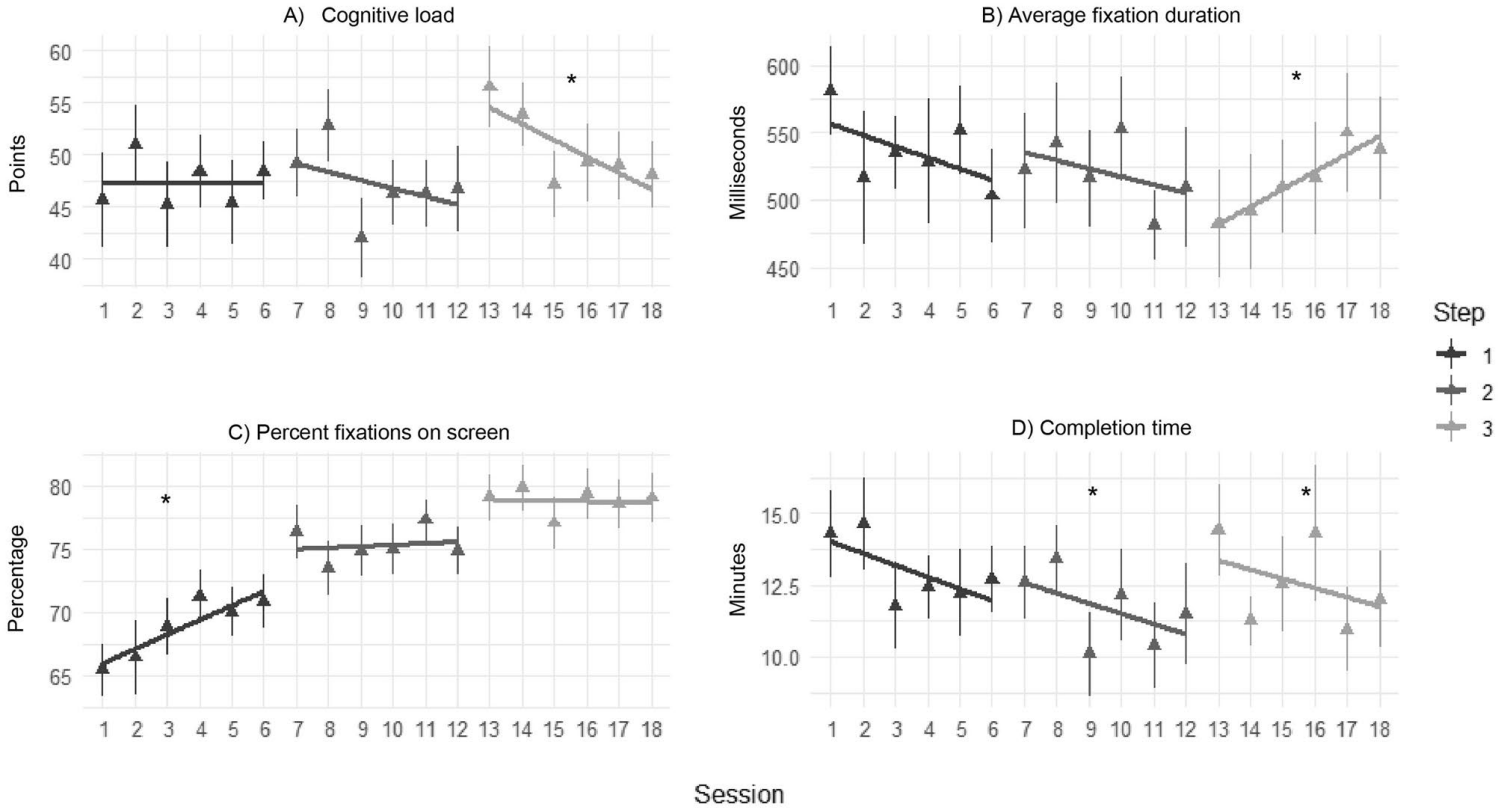
Development of dependent variables over training. Triangles depict the mean and vertical lines depict the standard error for the mean. The horizontal lines represent the linear effect of sessions. The star indicates a statistically significant development.

See Fig. 3 for an overview of the assessed variables. Completion time decreased within all steps, but only for step two and three the decrease was statistically significant. Step two: *F*(1, 41) = 5.38, *p* = 0.025; mean change per session =  − 0.04 (0.38 min), 95% CI: − 0.07 to − 0.01 (− 0.68 to − 0.08 min). Step three: *F*(1, 15) = 4.75, *p* = 0.046; mean change per session =  − 0.03 (0.32 min), 95% CI =  − 0.06 to − 0.003 (− 0.62 to − 0.03 min).

Both, cognitive load and average fixation duration showed statistically significant developments within the last step only. For cognitive load the mean decrease was 1.56 points per session, *F*(1, 57) = 10.80, *p* = 0.002, 95% CI: -2.05 points to -0.61 points. Fixation duration increased within the last step, *F*(1, 15) = 6.54, *p* = 0.022, mean change per session = 0.24 (15 ms), 95% CI: 0.006 to 0.48 (3 to 26 ms).

Amount of fixations on screen showed an overall increase over all 18 sessions, *F*(1, 14) = 100.01, *p* < 0.001, mean change = 0.77% (95% CI: 0.55% to 0.98%). Analysis of steps showed a clear increase within the first step, but not within the others, *F*(1, 76) = 11.65, *p* = 0.001; mean change per session = 1.15%, 95% CI: 0.48% to 1.80%.

### Further exploratory analyses

**Figure 4:**
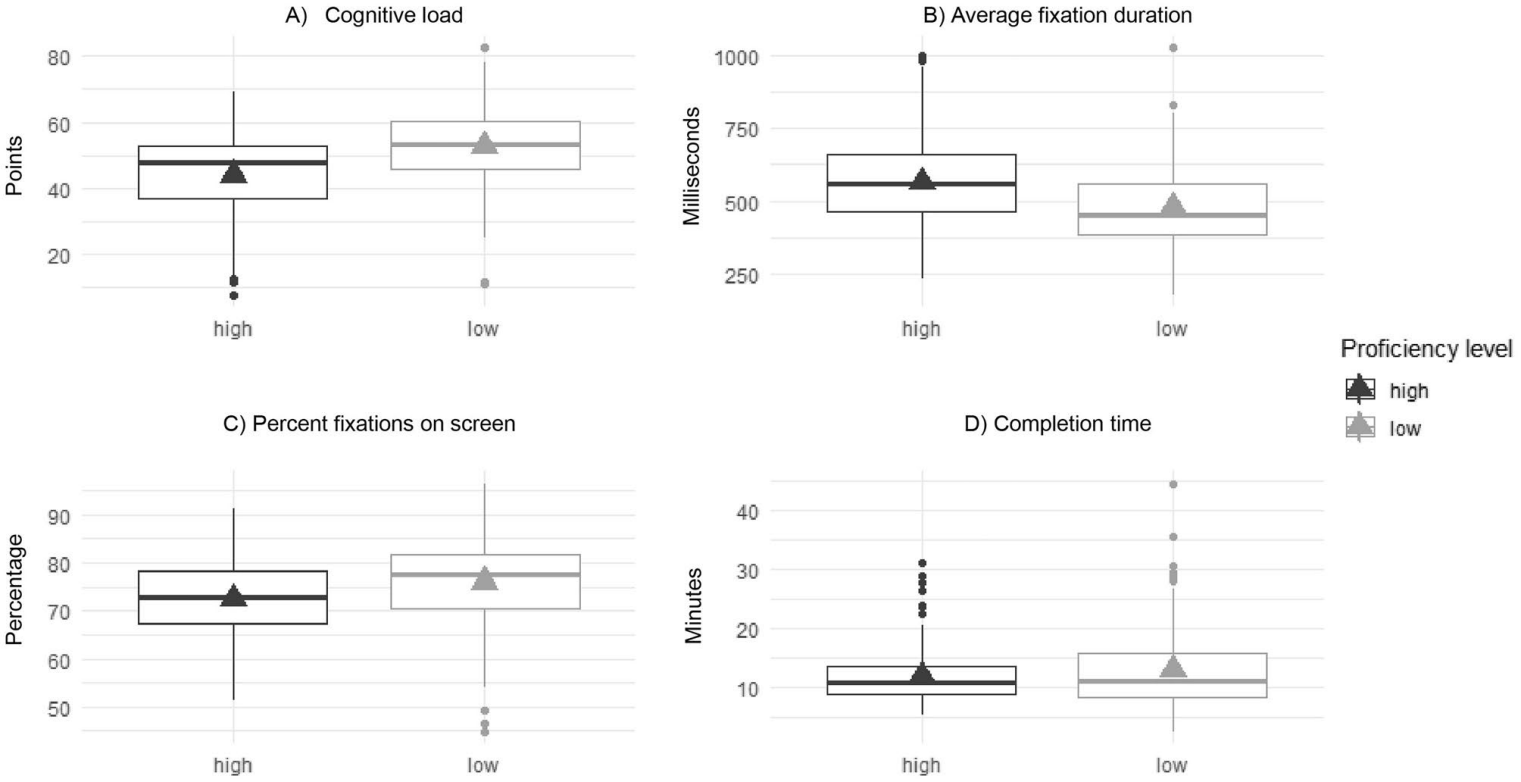
Differences in dependant variables between proficiency levels. Triangles depict the mean. Horizontal lines represent the median. The box depicts 50% of values (25th and 75th percentile) and the whiskers above and below the box mark the 90th and 10th percentiles. Points are outliers.

Based on their average global rating of all six sessions that were rated, we separated participants into proficient and less proficient surgeons, by means of a median split. If a participant’s value was below three points on average, they received the proficiency level “low” and above “high”. We then conducted Welch’s t test to compare the groups in terms of overall completion time, cognitive load, average fixation duration and percent fixations on screen. There were statistically significant differences in all variables except completion time. Proficient participants had lower cognitive load (95% CI: − 11.75 to − 5.46 points), higher average fixation durations (95% CI: 50 to 119 ms), and lower percentage fixations on screen (95% CI: − 5.59 to − 1.43%), see Fig. 4.

**Figure 5:**
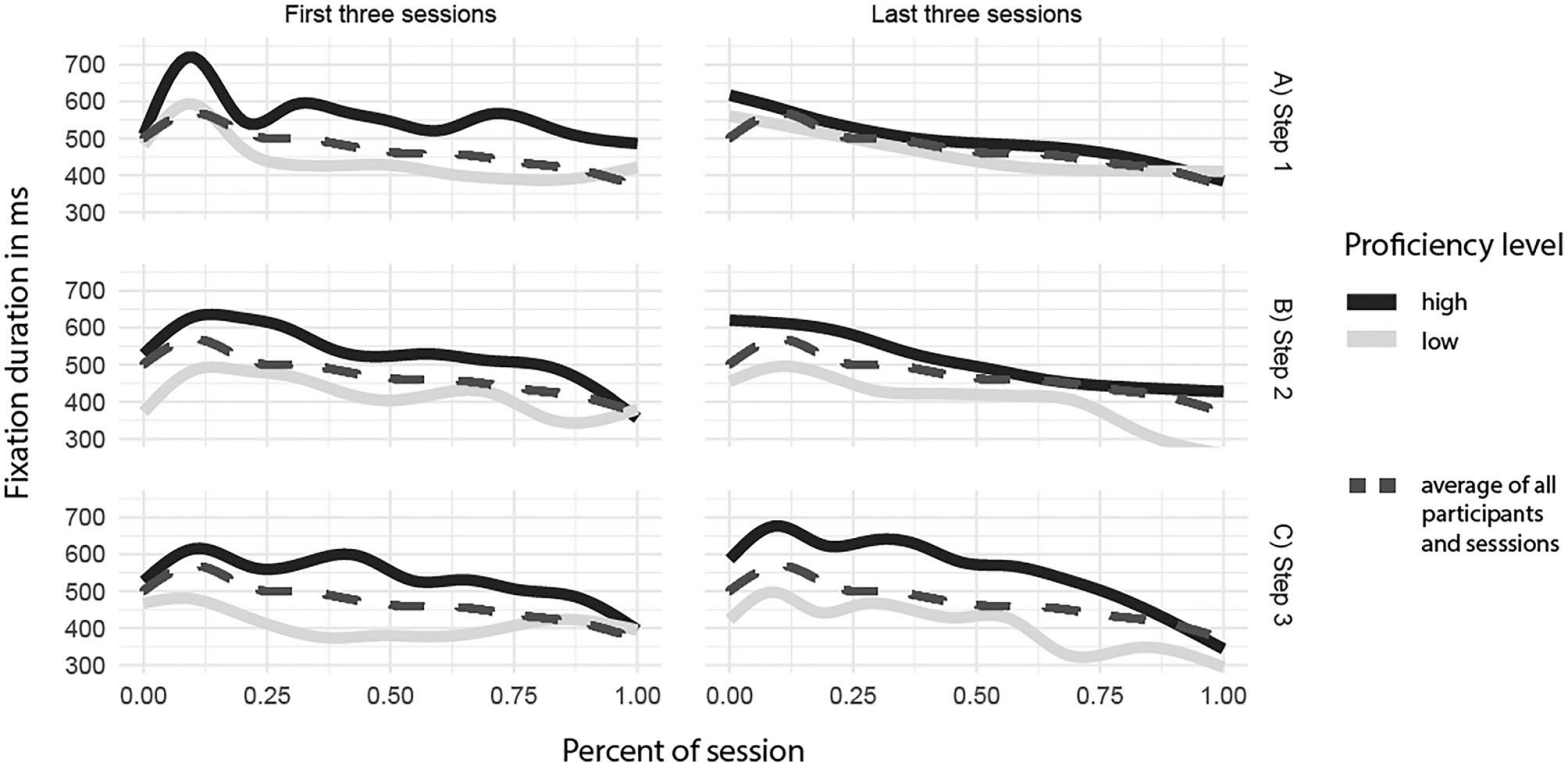
Development of fixation duration within first and last three sessions per step. Time is represented as percentage. The dotted line depicts the overall development averaged over all participants and sessions.

We also inspected the development of fixation duration within surgery sessions. Since completion time was different for each participant and session, we used percentage instead of time. Figure 5 represents the influence of proficiency level compared over the three steps, each divided into the first three sessions and the last three sessions. Average duration of fixations for all participants together (dotted line) increased in the beginning, peaked after 11.5% of the time, and declined afterwards. The peak was more elaborate in the first three sessions, especially during step one. Proficient participants had overall higher fixation durations and peaked on average 97 ms higher (Welch’s t-test: *p* < 0.001; 95% CI: 47 to 148 ms).

**Figure 6:**
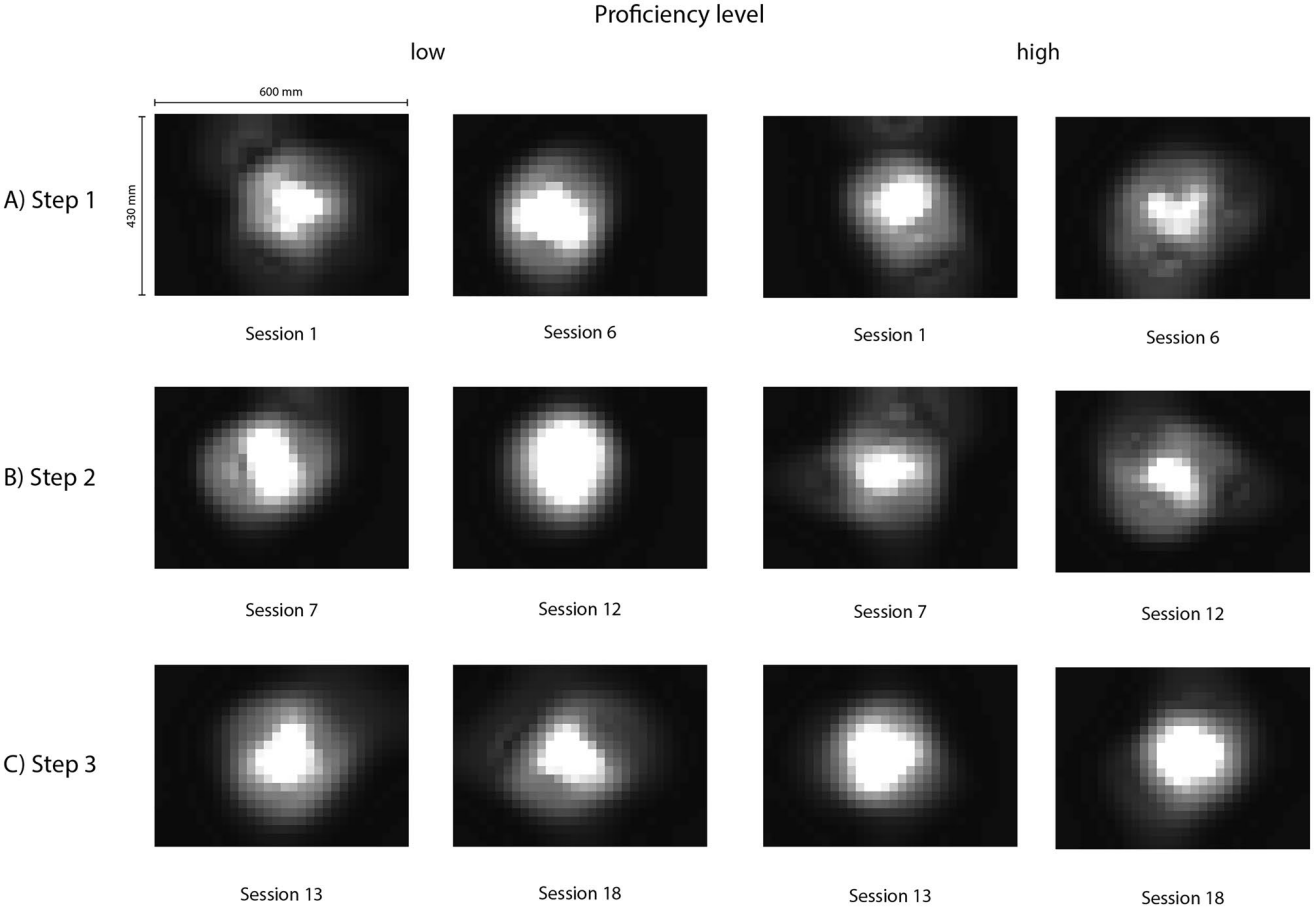
Grayscale heat maps for the first and last session of each step. Areas that have received more visual attention appear brighter. The term mm refers to millimetres.

An alternative way to inspect participants’ focus is the analysis of heat maps. Figure 6 shows the development of heat maps from the first session of each step to the last session of the step. Additionally, we separated between high and low proficient participants to point out possible differences. The heat maps show that the focus of proficient participants seems to be more diverse. Less proficient participants, on the other hand, had usually a larger area that was focused by more people.

## Discussion

We assessed OTO-HNS residents’ learning curve regarding FESS in terms of surgical performance, completion time, cognitive load, and eye movements over the course of 18 surgical sessions, split into three steps. Prior to every session, participants watched a standardized video of the surgery performed by an experienced consultant, half the participants also seeing the consultant’s gaze. We found no effect of gaze instruction except for a difference in surgical performance on OSATS rating in the first session. Dependent variables developed differently in the course of training. Analysis of participants’ surgical performance showed that average fixation duration was higher for residents with a high proficiency, whereas cognitive load and percent fixations on screen were lower.

### Effect of gaze instruction

Results indicate that gaze instruction did not influence the assessed variables. There was a difference in overall surgical performance at baseline, but the group with gaze instructions caught up and the remaining development was comparable. The difference in the beginning could be explained by an overstraining of the participants, with gaze instruction in addition to the demanding setup and tasks, so they first had to adapt. No statistically significant difference between groups was observed for the other dependent variables.

Previous studies showed the expert’s gaze during the surgery^16–18,33^, whereas in our study, the gaze instruction was shown before the surgery. The missing difference between groups could indicate that the transfer from the introduction video to the actual procedure did not work. However, our installation did not need the expert surgeon to be present during the training and would therefore allow residents to practice more independently. Alternatively, the surgery’s complex characteristics in the present study might explain the missing differences. Compared to the other studies, where single technical tasks such as picking and dropping balls were assessed, we measured a real surgical simulation. Other arguments could be the number of sessions or participants’ experience level. More research is needed to find the ingredients for an efficient and applicable gaze training.

### Changes of variables within steps

#### Completion time

The effect of the training program translated to a decrease in time needed to complete the surgeries after the initial six sessions. For step two and three a statistically significant decrease in the surgical time was observed, corresponding with previous research in FESS^34–36^.

#### Cognitive load and fixation duration

Average fixation duration and cognitive load showed a similar development throughout all sessions. They both remained the same within the first and second step, and then changed statistically significant within the last step. Whereas fixation duration increased, suggesting a more focused gaze, cognitive load decreased. This indicates that participants perceived the last step more mentally demanding than step one and two. The posterior ethmoidectomy can be considered more difficult than step one and two, mainly due to the proximity to the skull base. However, an increased focus during the last step might have helped participants decreasing their cognitive load to a level that was comparable to the other steps.

#### Percent fixations on screen

Percent fixations on screen increased during training and showed tendencies of a plateau in step two and three. In line with other studies^11,12,37,38^, participants looked more at the screen with increasing experience. They adapted their gaze from the second session on, showing a steep learning curve. Naturally, there needs to be a plateau effect, as surgeons have to look at their tools. Especially in the current setting, since participants had to take the tools themselves from the table. Comparison with training studies from Wilson et al.^12^ and Vine et al.^33,37^ suggest that participants attained a sufficiently expertise-like gaze strategy in step two with at least 75% of their gaze located on the target. This indicates that gaze expertise was reached before 18 sessions of training in our cohort.

#### Comparison between proficiency levels

Examination of different proficiency levels revealed no difference between completion time, whereas the other variables were able to distinguish between the two levels. Completion time is often used as an indicator of skill level; however, it should always be used in comparison with other variables^39^. Our results confirm this proposal and suggest that the sole application of completion time is precarious and not sensitive enough, especially when comparing surgeons of similar experience. The differences in cognitive load and fixation duration are in line with previous research^13,38^. The difference in percent fixation on screen are low but indicate that there might be an optimum of percentage distribution between target and tools, which still needs to be investigated.

Development of fixation duration within the surgery showed a clear pattern of the overall curve, indicating that participants first have a look around but then show a much-focused gaze. This could indicate a slowing down process as framed by Moulton et al.^9,40,41^. Slowing down refers to the cognitive transition process when a task becomes more difficult or effortful and therefore needs a more focused cognitive state. Results from all sessions indicate that residents slowed down their gaze after a first inspection to focus on the surgical task during the surgery. This also goes in line with results by Vickers’ research group^42,43^ concerned with the quiet eye research. Their results state that a strong focus during significant situations in a surgery can be trained and leads to better surgical outcomes.

Further analyses reveal that the overall curve was mainly shaped by the first three sessions of a step as compared to the last three sessions. Especially during the first step, the medialisation of the middle turbinate along with the resection of the uncinate process led to a significant peak in fixation duration. This peak may be explained by the first incisions of the training course. The close observation on how the model behaves and how the artificial tissue should be handled are possibly the cause for this observation in eye movements.

Proficient participants had overall higher fixation durations and peaked higher. This is in agreement with previous findings^13,14^ and further evidence for the information-reduction hypothesis in minimally invasive surgery^44^. This theory states that surgeons with more expertise are less distracted by task-redundant information due to their selective perception. Therefore, they show a more focused gaze compared to surgeons with less expertise. Analysis of heat maps did not show the same pattern, since less proficient participants seemed to look more at the same place, what could indicate a focused gaze. However, due to the applied setup, camera handling heavily influences this measure. Therefore, the differences visible in the heat maps could also be due to individual differences and preferences in camera handling. Whereas low proficient participants might have stuck to the shown standard procedure, high proficient participants might have adapted their handling, which resulted in more diverse camera positions and thus a more distributed focus.

### Limitations

Despite the high ecological validity, the current study has several limitations. First, we did not apply baseline testing before assigning participants to their respective group, which could have uncovered pre-existing differences in participants’ abilities. Second, since we did not perform a manipulation check, we can only assume that participants were affected by the gaze instruction. Lastly, there was not enough data for more sophisticated analyses, which could provide further insights. However, with 16 residents and each with 18 measuring points, the current study provides robust and useful information.

## Conclusion

Analysis of eye movements over the course of 18 surgeries showed significant increase of percent fixations on the screen. Compared with previous research in similar settings^12,33,37^, residents in this study applied an expert-like gaze strategy after half of the training sessions. Moreover, an increased focus in terms of fixation duration might have helped residents to reduce cognitive strain during posterior ethmoidectomy. Furthermore, average fixation duration can distinguish between proficiency levels and the development of fixation duration within sessions follows a specific schema. Therefore, eye movements in FESS provide insightful objective information on surgeons’ performance.

## Supplementary Information

Supplementary Information.

## Data Availability

All data is available by the corresponding author upon request.
