# Supplementary material for: Training and proficiency level in endoscopic sinus surgery change residents’ eye movements
Source: Sci Rep. 2023 Jan 3;13:79. doi: 10.1038/s41598-022-25518-2 (PMC9810736; doi:10.1038/s41598-022-25518-2)
Supplement: Supplementary file 1 — Supplementary Information. [file 41598_2022_25518_MOESM1_ESM.docx]

# Supplemental analysis

There was no statistically significant difference between the two groups (*F*(1, 14) < 1.80, *p* > 0.20). eFigure 1 shows the development of the two groups over the entire training.

eFigure1. Development of two groups over training


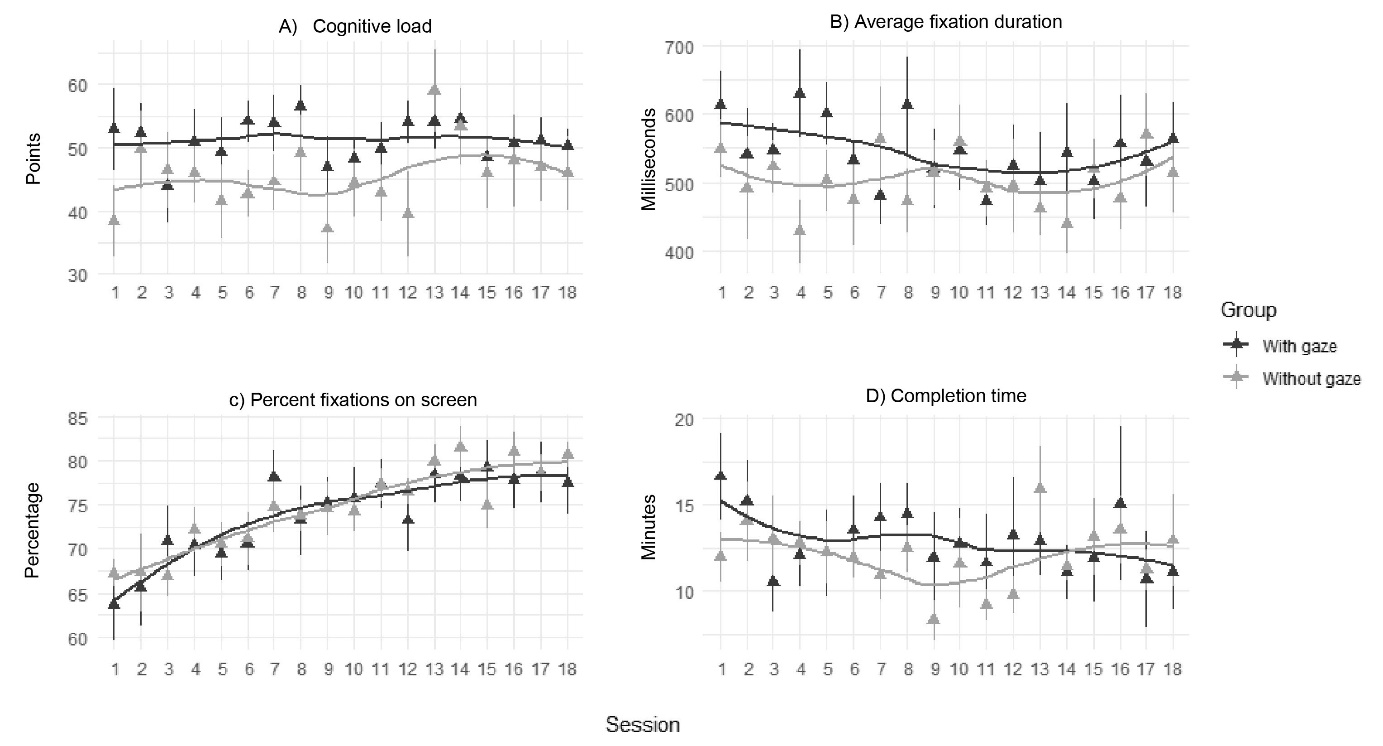


*Note*. Triangles depict the mean and vertical lines depict the standard error for the means per session. Horizontal lines depict the smoothed development over all sessions (local regression).

eTable 1. Correlation between dependant variables.

|  | Global rating (OSATS) | Time | Cognitive load | Fixation duration | Percent fixations on the screen |
| --- | --- | --- | --- | --- | --- |
| Global rating (OSATS) | 1.0* |  |  |  |  |
| Time | -0.19 (-0.38 to 0.006) | 1.0* |  |  |  |
| Cognitive load | -0.11 (-0.30 to 0.09) | 0.28* (0.17 to 0.38) | 1.0* |  |  |
| Fixation duration | 0.07 (-0.12 to 0.28) | -0.02 (-0.13 to 0.10) | -0.16* (-0.28 to -0.05) | 1.0* |  |
| Percent fixations on the screen | 0.05 (-0.16 to 0.24) | 0.09 (-0.03 to 0.20) | 0.36* (0.26 to 0.46) | -0.52* (-0.60 to -0.43) | 1.0* |

*Note*. Pearson correlation with respective confidence interval in brackets. The star indicates a statistically significant correlation.
